# Barriers to Overcoming Child Hunger and Malnutrition: Applying a Human Rights Approach to Improve Policy and Action

**DOI:** 10.3389/ijph.2023.1605969

**Published:** 2023-08-30

**Authors:** Carolina Mejía Toro, Angela Carriedo, Eliana Maria Pérez Tamayo, Eric Crosbie

**Affiliations:** ^1^ Department of Noncommunicable Diseases and Mental Health, Pan American Health Organization/World Health Organization, Washington, DC, United States; ^2^ Bureau for Humanitarian Assistance, United States Agency for International Development, Washington, DC, United States; ^3^ World Public Health Nutrition Association, Peacehaven, United Kingdom; ^4^ Department of Health, University of Bath, Bath, United Kingdom; ^5^ School of Nutrition and Dietetics, University of Antioquia, Medellín, Colombia; ^6^ School of Public Health, University of Nevada, Reno, Reno, NV, United States; ^7^ Ozmen Institute for Global Studies, University of Nevada Reno, Reno, NV, United States

**Keywords:** policies, child, hunger, malnutrition, food security

## Abstract

**Objective:** Analyze key barriers to achieving children’s right to food under Colombia’s food and nutrition security policies and programs.

**Methods:** A literature review was conducted along with 17 semi-structured expert interviews. The law framework on the right to food was applied to analyze findings.

**Results:** Four key barriers were found. First, a reductionist approach prevails in the political narrative. This focuses on ensuring personal food access overlooking societal and environmental impacts. Second, the implementation of policies and programs is passed on to third parties, preventing civic participation and accountability. Third, there are insufficient national data sources and indicators to monitor the impact of interventions and funding. Fourth, program implementation is unequal and inadequate, which inadvertently supports illicit economies that thrive on conditions of hunger and poverty.

**Conclusion:** Children’s food and nutrition are reliant on organizations that focus on personal food supply without strengthening civic participation. Strengthening participation requires a human rights approach. International organizations can help the government to engage communities in policy and program improvement and oversight.

## Introduction

A large body of evidence shows that hunger and malnutrition, mainly during early stages of life, increase the overall risk of disease and cause irreversible cognitive, psychological, growth, and immunological impairments [1–6]. These effects are intergenerational and create disadvantages compared with nourished peers in terms of access to opportunities and equal outcomes [1–3, 6, 7]*.* The Food and Agriculture Organization of the United Nations (FAO) defines hunger as an uncomfortable or painful sensation caused by insufficient consumption of dietary energy [8], and malnutrition as the result of deficiencies or imbalances in energy and nutrients consumption. Malnutrition can result from the lack of regular access to sufficient, safe and nutritious food for normal growth, development, and an active healthy life, or from inappropriate childcare practices, insufficient health services and unhealthy environments [8].

To combat these issues, governments and international organizations have adopted policies and programs aimed at promoting food security [9–12]. Food security is the condition where every person has permanent access to sufficient, safe and nutritious food to meet their dietary needs and preferences for an active and healthy life [8]. To promote food security, governments have adopted policies that seek to improve a population’s access to food [8–13] by increasing market staples through intensive agriculture, manufacturing, and imports [10–12]. Parallelly, government and international non-governmental organizations (INGOs) distribute handouts to households that cannot economically access sufficient staples [10–13]. However, these efforts seem insufficient. Although total food production is enough to feed everyone [14], 29.3% of the global population cannot access a healthy diet, including 8.9% of children under five suffering from hunger and 3.8% impacted from some type of malnutrition [8, 15].

Recognizing limitations in food security-focused approaches, scholars and law experts have proposed grounding food and nutrition policy and programs on human rights, rather than on a food security aim [10–13, 16–18]. This is because international human rights law recognizes adequate food as a human right essential for the enjoyment of all human rights and establishes clear correlated obligations [16–23]. The right to adequate food (RtAF) is enshrined in the Universal Declaration on Human Rights (UDHR) Article 25 [19], the International Covenant on Economic, Social and Cultural Rights (ICESCR) Article 11 [20], the Convention on the Rights of the Child (CRC) Article 24 [21], and several other legal instruments [22]. Moreover, General Comment No. 12 of the United Nations Committee on Economic, Social and Cultural Rights (CESCR) (GC12 hereafter) defines the RtAF as the right of every person, alone and in community with others, to have permanent physical and economic access to adequate food or means for its procurement [22]. GC12 establishes correlative state obligations to respect, protect, and fulfill the RtAF, which are not about charity, but about ensuring that everyone can feed themselves in dignity [18, 22]. Conversely, no international law instrument defines food security or establishes correlated state obligations [23]. Nonetheless, evidence shows that legal recognition alone is not enough to ensure the RtAF [10–17] so states must take every necessary action to bring formal recognition into practice [22].

The Latin America and the Caribbean (LAC) region is one of the worst regions in terms of hunger and malnutrition figures that involve the insufficiency of ongoing food security measures to ensure the RtAF [12]. In LAC, 40.6% of people of all ages do not have the means to follow a healthy diet, while the global average is 29.3%. Moreover, 56.6 million people suffer from hunger, which increased 30% between 2020 and 2021, the highest increase globally [8, 24, 25]. Within LAC, Colombia has the highest rate of hunger and malnutrition in the region, where 54.2% of people are unable to access a healthy diet and 20.6% of children under five suffer from some form of malnutrition [24–27].

The Colombian rates of child hunger and malnutrition are especially striking [26–28] although the Colombian regulatory framework provides special protection to children and their RtAF [29, 30]. The 1991 Colombian Political Constitution Article 44 establishes the RtAF as a fundamental right of children and upholds UDHR, ICESCR, and CRC [29]. Accordingly, the Colombian government has established policies and programs to protect the RtAF [17, 30]. However, previous research has identified barriers that impede their efficacy, including: 1) inconsistencies between national food and nutrition policies and the rest of the social, economic, agrarian, and foreign policies [12, 31]; 2) lack of coordination between the institutions in charge of implementing food and nutrition policies and the bodies in charge of national and subnational budget administration [31–35]; 3) impact of the armed conflict and insecurity [10, 31]; 4) inequity in the distribution of land and production means and [11, 31] 5) lack of continuity in the political commitments from one mandate to another [31, 35]. Still, little research has explored why despite having policies and programs framed under the RtAF and children protections, Colombia is not ensuring children’s RtAF, and addressing child hunger and malnutrition relief in practice.

This article aims to fill that gap by identifying and analyzing key barriers to achieving children’s RtAF under Colombia’s existing food security and nutrition policies and programs. Using ICESCR Article 11 and G12 as a framework to analyze our results, we explore how barriers materialize due to a lack of mechanisms that ensure civic participation and monitor the resources and outcomes of policies and interventions. Understanding these barriers can strengthen Colombia’s food and nutrition decision-making and further inform other countries.

## Methods

### Literature Review

Between May and August 2021, we conducted a literature review that included peer-reviewed and non-peer-reviewed articles. We used Google, Google Scholar and searched Ebsco, Elsevier, and PubMed databases using key search terms: “hunger OR malnutrition AND human rights,” “state of the right to food,” “right to food AND policy AND Colombia.” For the first two combinations of search terms, we did not include the key word Colombia to allow search results related to both international and Colombian peer-reviewed articles, INGO reports, government documents, human rights law, academic reports, and international law handbooks. For the third combination, we included the key word Colombia to focus on Colombian documentation. We did this considering the international context exerts influence on Colombia’s food and nutrition status and legislation, but the focus of the study was Colombia’s national policy framework. We found 138 documents, of which 77 were relevant to our study. Twenty-eight of them were peer-reviewed articles, 15 INGO reports, 12 government documents, 12 legislations on human rights, 7 university reports, and 3 international law handbooks. We included only publications in English and Spanish. Furthermore, we did not use an exclusion criterion for publication dates, considering that relevant reports, law, and policy documents would probably have been published or adopted on different dates, even with notable differences between them. Moreover, documents which could be considered old or out-of-date, could be key to understanding more recent documentation.

### Semi-Structured Interviews

Between June and August 2021, we sent email invitations to 18 experts in food and nutrition law and policy. The purpose of the interviews was to identify barriers that prevent food and nutrition policies and programs from effectively addressing child hunger and malnutrition. We conducted expert semi-structured interviews to collect insights of people with first-hand knowledge on the topic. This allowed us to further explore the main limitations and strengths of the policymaking that are minimally reflected in policy documents or in public data [36]. Participants were identified through peer-reviewed articles, using standard snowball sampling [37]. In total, 17 participants agreed to be interviewed, including two government officials, six international organization experts, three civil society actors, and six academics. Interviews were conducted in Spanish [16] and English [1]. Interviews lasted between 30 and 120 min and were based on a semi-structured interview guide (see Supplementary File S1). Each interviewee signed an informed consent form following the European Standards of Ethics in social science research. Conversations were recorded, transcribed, and analyzed through thematic analysis [38]. Transcription and analysis were done in MAXQDA^®^, a software for qualitative and mixed methods data analysis.

### Analytical Framework

We analyzed the interviews through deductive and iterative analysis, using the legal framework on the RtAF [19–22]. Findings are presented as a narrative synthesis of the key barriers identified.

### The Right to Food

**FIGURE 1:**
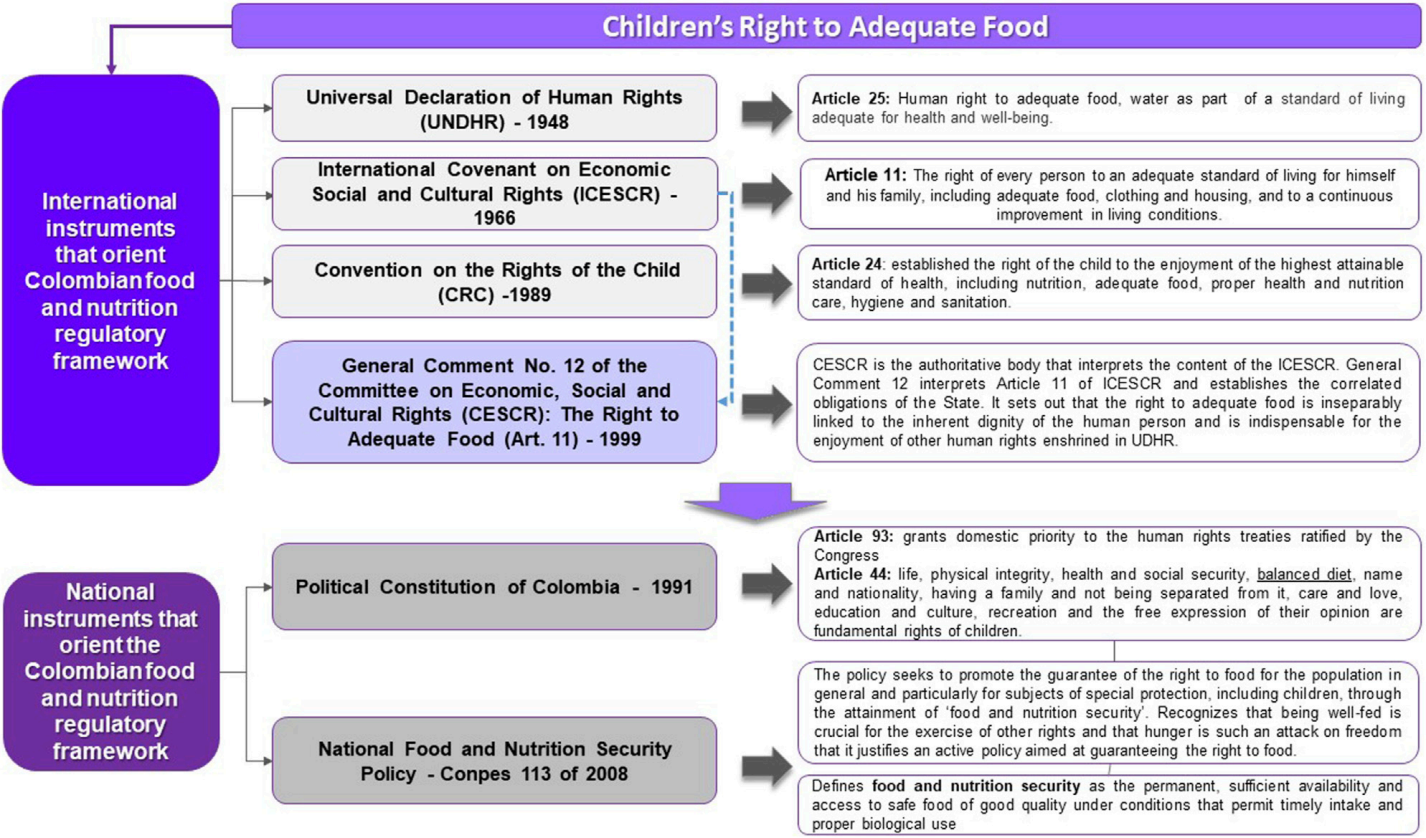
Analysis framework of the right to adequate food for children (Policy Solutions to Realize the Right to Food of Children in Colombia: An Integrative Approach, Colombia, Italy, 2021–2022).

We approached the RtAF as enshrined in Article 11 of ICESCR [20] and interpreted by GC12 [22]. G12 establishes that ICESCR member states are bound to undertake all necessary measures for the progressive realization of the RtAF without discrimination, including international cooperation [24]. Having ratified ICESCR, Colombia is a member state and abides to GC12 [29, 39]. To comply with its obligations towards the RtAF, Colombia established the National Policy on Food and Nutrition Security Conpes 113/2008, which promotes “food and nutrition security” as the way to guarantee the RtAF [30] (see Figure 1). Applying this framework, we examined the documents and interviews to understand key barriers to achieving children’s RtAF under Colombia’s food security and nutrition policy and programs.

## Results

### Barriers

We identified four key barriers to achieving children’s RtAF under Colombia’s food security and nutrition policy and programs; 1) a reductionist approach to improving nutrition; 2) policy and program implementation passed on to third parties; 3) insufficiency of national source data; 4) uneven and inadequate program implementation.

### Barrier 1: Reductionist Approach to Improve Nutrition

#### National and International Focus on Food Security

As of February 2023, all food and nutrition policies in Colombia were based on Conpes 113/2008 [33]. Conpes 113/2008 coined the term “food and nutrition security,” drawing on the 1996 World Food Summit (WFS) definition of food security [9, 33]. The 1996 WFS definition has oriented food and nutrition programs of many governments and INGOs and, as all interviewees reported, guided the initiatives for hunger and malnutrition in Colombia. However, neither Article 11 of ICESCR nor GC12 mentions food security as equal to or part of the RtAF, nor does it establish state obligations correlated with food security [20, 22, 23]. Several interviewees saw this as an important barrier and a law expert stated, “because food security is not a legal concept, it does not imply clear obligations to the state or stakeholders, leaving its interpretation and implementation to the discretion of politicians and organizations” (see Table 1).

**TABLE 1:** Assessment of the four main barriers to achieving children’s right to adequate food under Colombia’s existing food security and nutrition policy and programs (Policy Solutions to Realize the Right to Food of Children in Colombia: An Integrative Approach, Colombia, Italy, 2021–2022).

ICESCR Article 11 and GC12 standards	Application of ICESCR Article 11 and GC12 to Colombia food policies	Barriers to guaranteeing children’s right to adequate food according to ICESCR Article 11 and GC12
Barrier 1. Reductionist approach to improving nutrition		
Focus on food security		
• No legal definition of food security	• Conpes 113/2008 guides all food and nutrition public policies in Colombia	• Food security is not a human right protected under international law
• No mention of food security as a means to achieve the right to adequate food	• WFS 1996 food security definition orients Conpes 113/2008 and the majority of INGO programs	• Food security concept lacks clear correlated legal obligations
• No clear state obligations on food security	• Food security is approached as equivalent to the right to adequate food or the path to achieve it	• Food security remains open to interpretation and is susceptible to manipulation and instability
Barrier 1. Reductionist approach to improving nutrition		
Focus on personal food access		
• State obligation to *Respect:* refraining from any measures that impede the right to adequate food for any person or human group • State obligation to *Protect:* taking measures to ensure that no entity deprives people of adequate food or the means to access it (e.g., land, ecosystems)	• Conpes 113/2008 and WFS 1996 focus on ensuring food access at personal level	• Intensive farming, manufacturing and imports endanger small economies and ecosystems
	• Focus on individual persons overlooks possible impact on communities and ecosystems	• Results in national food dependance from foreign suppliers and corporations
	• Attempt to ensure personal food access through	• Small entrepreneurs, peasants, and Indigenous communities are deprived of means to access adequate foods
	⁃ Augmenting market staples by means of mass production and imports	• They fall into poverty and depend on handouts
	⁃ Handouts for people unable to obtain sufficient food from the market	• State failure to *protect* and *respect*
Barrier 1. Reductionist approach to improving nutrition		
Resolution as charity		
The right to adequate food should not be interpreted in a narrow sense as handouts or packages of calories or nutrients	• Government and international organizations approach the resolution of hunger and malnutrition as increasing food supplies at the personal level	• Handouts and supplementation may provide calories and nutrients that reduce hunger and malnutrition rates
	• To mitigate access constraints, they distribute handouts	• However, the causes of hunger and malnutrition remain unsolved
	• Handouts do not address the structural causes of food shortage and malnutrition	
Barrier 2. Policy and program implementation passed on to third parties		
Outsourcing favors corruption		
States should provide an environment where private businesses functions, in respect to the right to adequate food and compliance with the principles of accountability and transparency	• Government and international organizations hand program implementation over to third parties	Passing implementation to third parties impedes accountability and transparency:
	• Most implementing parties are private food, beverage and healthcare companies	• By creating distance between responsible organizations and recipients, impeding social accountability
	• Outsourcing program implementation increases the risk of: politically favorable and corrupt contracts to tenders, misappropriation of public resources, embellishment of public figures, and delivery of low-quality, ultra-processed, rotten, or expired foods	• Because suppliers are motivated by profit over compliance with human rights
		• The state is not ensuring conditions for suppliers to operate with accountability, transparency, and respect for the right to adequate food
Barrier 2. Implementation of policies passed on to third parties		
Outsourcing hinders civic participation		
The State must guarantee the principles of people’s participation and decentralization in every food and nutrition policy and program	• Program implementation shifts from supplier to supplier and tender to tender, creating miscommunication between organizations and program recipients	• State has not planned programs in a way that ensures participation throughout the full policy and program cycle (formulation, implementation, monitoring, evaluation)
	• Recipients cannot communicate problems and propose solutions	
	• Communities develop functional solutions to their food and nutrition problems	• Resolving this is essential to comply with the principles of participation and decentralization
	• Miscommunication impedes solutions to be known outside the communities	
	• Reduced opportunities of investment to upscale solutions and divulge as best practices	• Supporting community solutions can complement the efforts of the state and the international community
	• Hindered understanding of citizens as right holders instead of aid beneficiaries	
Barrier 3. Insufficiency of national source data		
The State must comply with the principles of accountability, transparency and people’s participation	• ENSIN has inconsistent indicators from one survey to the following	• Lack of timely food and nutrition data to oversee and evaluate the implementation and impact of policies and programs
	• ENSIN is only conducted every 5 years and is often delayed	• Lack of information hinders transparency and accountability
	• ENSIN lacks specific indicators to measure the impact of food and nutrition policies and programs	• Integrating mechanisms for citizen participation and oversight is needed to
	• No system to collect national data by asking recipients about their experiences, problems, and suggestions	⁃ Identify functional and dysfunctional interventions and avoid repeating mistakes
		⁃ Derive evidence-based strategies
		⁃ Achieve accountability and transparency
Barrier 4: Uneven and inadequate program implementation		
• State obligation to *Respect:* refraining from any measures that impede the right to adequate food for any person or human group. • State obligation to *Protect:* taking measures to ensure that no entity deprives people of adequate food or the means to access it (e.g., land, ecosystems)	• Unequal, inadequate distribution of handouts	• The inequality and inadequacy of aid distribution violates the state’s obligation to *respect* the right to adequate food
	• Handouts do not reach many remote territories, most of which are affected by armed conflict and state absence	
	• Uneven distribution reinforces pre-existing inequities	• Handouts do not solve the causes of poverty, hunger, malnutrition, and the links with illicit economies
	• Some handouts consist of expired, culturally inadequate, highly processed, energy-dense foods, manufactured by multinational corporations, threatening public health and local economies	
	• People in territories affected by poverty, hunger, malnutrition and state absence are vulnerable to engage in illicit economies	• Lack of forceful measures shows a clear violation to the obligations to respect and protect the right to adequate food
	• Other than the inadequate handouts, the state has not taken measures to intervene	

#### Focus on Personal Food Access

GC12, paragraph 15, establishes that ICESCR state parties are obliged to: 1) *respect* the RtAF by refraining from any measures that impede this right to any person or human group; 2) *protect* the RtAF by taking measures to ensure that organizations, companies, or individuals do not deprive people of adequate food or the means to access it (e.g., land, ecosystems) [22]. All interviewees reported that food security and food and nutrition security focus on guaranteeing personal food access. Some interviewees commented that this individualized focus does not consider whether the ways to supply individuals with food may negatively impact communities and ecosystems. For example, the Colombian government attempts to augment food access by increasing mass food production and imports. Parallelly, the government and INGOs distribute handouts to individuals considered the most vulnerable in society. These approaches increase food supplies. Yet, they disregard that intensive farming and imports favor corporations, big landowners and manufacturers while endangering ecosystems, small-scale farms and businesses, producing food dependency. Furthermore, assistance programs are not coupled with strategies to ensure that people have the means to decide and access to adequate food on their own. According to one interviewee, this reveals that the State is not taking measures to prevent corporations and organizations from depriving certain vulnerable groups (i.e., indigenous groups, remote communities, areas affected by the armed conflict) of the means to procure adequate food and cause food dependency on industrial production and assistance programs.

#### Resolution as Charity

GC12, paragraph 6, underlines that RtAF should not be interpreted in a narrow sense as packages of calories or nutrients. All interviewees aligned with GC12, underscoring that a prominent barrier to having efficient food and nutrition policies is the “frequent interpretation by government and international actors of the resolution of hunger and malnutrition as charity” or “nutrition supplementation” focused on disproportionally affected individuals. These observations suggest a widespread paradigm that reduces action on food and nutrition to handouts for specific populations, instead of understanding adequate food as a human right and an obligation of the state under international law. According to one interviewee, “While handouts may reduce malnutrition rates, handouts cannot solve the causes, so malnutrition persists.”

### Barrier 2: Program and Policy Implementation Passed to Third Parties

#### Outsourcing Favors Corruption

GC 12, paragraphs 20 and 23, respectively, establish that states should provide an environment where private business pursues its activities within a code of conduct conducive to respecting the RtAF, and compliance with the principles of accountability and transparency. In this regard, all of the interviews revealed that government organizations and a majority of INGOs implementing food security and nutrition aid programs, hand their implementation over to third parties, most notably to food, beverage and healthcare providers. According to the interviewees, outsourcing program implementation impedes compliance with the principles of accountability and transparency by increasing the probability of corruption. This includes politically favorable and corrupt contracts to tenders, misappropriation of public resources, embellishment of public figures, and suppliers cutting costs by delivering low-quality, ultra-processed, rotten, or expired foods, despite being paid enough for adequate food. Some interviewees revealed that, in order to continue receiving international cooperation funds for food and nutrition, which represent important funding resources, the government must justify the allocation of these resources by providing evidence to donors on how they are invested. As evidence, the Colombian government shows reductions in national child malnutrition figures from the National Food and Nutrition Status Survey (ENSIN) [26]. One interviewee revealed that “to make food and nutrition programs appear to have a positive impact on ENSIN, government officials command hospitals in remote areas not to report malnutrition as a cause of death, when death can be attributable to other causes.” Another recurring practice to enhance ENSIN results is to “modify its indicators from survey to survey.” By modifying indicators, ENSIN results vary, thereby embellishing the numbers.

#### Outsourcing Hinders Civic Participation

GC12, paragraph 23 and ICESCR, Article 13 establish the obligation of states to guarantee civic participation. Several interviewees explained that outsourcing the implementation of food and nutrition aid creates distance between the responsible organizations and the recipients, as program implementation shifts from supplier to supplier. This prevents recipients from reporting problems and proposing solutions to decision-makers who develop and implement the policies and programs. Interviewees observed that this distance impedes the perception of citizens as right holders rather than aid beneficiaries. This perception is essential to achieve community participation and people’s sovereignty to decide on the food system [10, 11].

Four interviewees highlighted that despite these adverse circumstances, numerous communities across the country develop functional solutions to their food and nutrition problems (i.e., peasant networks, seed networks, and family markets). Nonetheless, due to the lack of communication between citizens and decisionmakers, many community solutions remain unknown to stakeholders who could mobilize resources to help grow and spread best practices in other communities. Experts agreed that INGOs can call upon the government to ensure participation in all policies and programs and attract resources toward functional community solutions.

### Barrier 3: Insufficiency of National Source Data

Applying GC12 and ICESCR principles of accountability, transparency, and participation helps to identify the limitations of available national source data on food and nutrition in Colombia. As of February 2023, ENSIN, last updated in 2015, remains the only national source data on food and nutrition in Colombia [25]. The interviewees agree that ENSIN has inconsistent indicators from survey to survey, is only conducted every 5 years, is often delayed, and lacks specific indicators to measure the impact of food and nutrition policies and programs. In fact, Colombia has no system to periodically collect national data on policy and program outcomes by asking recipients about their experiences, problems, and suggestions [33–35]. All interviewees observed that ensuring civic participation in data collection and program monitoring is the most efficient way to improve the quality and transparency of national data sources and to enhance program accountability. As one government official put it, “coordination between organizations and civil society at the national and subnational levels is essential to identify the initiatives perceived as functional and dysfunctional by the recipients, develop efficient evidence-based strategies, and avoid repeating mistakes.” These primary data converge with the results of comparative analyses of food and nutrition policies in Colombia [34]. The analyses found insufficient timely food and nutrition data to monitor and evaluate the impact of policies and programs. These studies reiterate the need for civil society, especially the recipients of interventions, to participate in decision-making, data collection, and oversight. Such participation requires a system of data dissemination that allows the government, involved organizations, and citizens (including civil society and oversight organizations, media, academia) to know the data, provide policy and program implementation feedback, and monitor resource allocation and use.

### Barrier 4: Uneven and Inadequate Program Implementation

In line with the content of GC12, paragraph 15, several interviewees reported that the distribution of food aid in remote territories is unequal and inadequate, thus violating the state’s obligation to *respect* the RtAF. The interviewees agreed that the assistance from national and international organizations does not reach many remote territories, which have experienced a history of armed conflict and state absence. These results align with previous research [35] that reveals an unequal implementation of food and nutrition policies and interventions between territories, without coordination with national and subnational development programs. The unequal distribution of aid ends up reinforcing pre-existing inequities between territories. Moreover, the interviews reveal that the aid that reaches some of the remote territories often consists of low quality, expired, and culturally inadequate foods that are often highly processed, energy-dense, and manufactured by transnational corporations. As a result, weak state intervention threatens public health and local economies, and makes public programs more prone to looting.

Simultaneously, as some interviewees reported, state absence combined with the high levels of poverty, hunger, and malnutrition in most remote areas in Colombia, often trigger people to resort to illicit activities. An interviewee exemplified that the government invests massive resources to combat narcotics. However, many production centers and transport routes are known, yet no forceful measures are taken. Decision makers “are aware that people in neglected territories affected by poverty, hunger, and malnutrition are vulnerable to engaging in illicit economies, such as narcotics, in search of survival. They are pressured by armed groups.” However, “in these territories it is not convenient to solve state absence with structural measures to address poverty, hunger, and malnutrition… Handouts serve to keep things as they are while pretending that something is done… Resources can be looted and allow the perpetuation of profitable illicit activities that benefit some people of the political elite while keeping their implication unclear and unpunished.” These results suggest that investments meant for food and nutrition handouts, may inadvertently support illicit economies that thrive in hunger, malnutrition and poverty, hampering the obligation to respect the RtAF.

## Discussion

This study provides important insights into what barriers impede the attainment of children’s RtAF under Colombia’s current food security and nutrition policy and programs. We describe the importance of designing, implementing, monitoring, and evaluating policies and programs in compliance with the obligations of the state toward the RtAF, acquired through ratification of ICESCR and CRC. In doing so, our findings reveal four critical barriers: 1) a reductionist approach to improving nutrition prevailing in the national political narrative; 2) policy and program implementation is passed to third parties, creating distance between the responsible organizations and the recipients; 3) insufficiency of national source data to assess the results of interventions’ outcomes and 4) uneven and inadequate implementation of aid interventions in remote communities, which inadvertently reinforces preexisting inequities and supports illicit economies that thrive in conditions of hunger and poverty. Although our study focused on children, findings suggest that these barriers affect both children and the entire community around them, including adult women and men. However, the implications of the barriers on human rights are greater for children, since childhood is a critical developmental window that defines, to a critical extent, lifetime access to opportunities and results, with effects on future generations [1–7]. This raises important implications for policy and program development when seeking to reduce child hunger and malnutrition, a problem that remains critical in Colombia, LAC, and most low- and middle-income countries [8, 24].

The way in which food and nutrition policies are framed is crucial to their success [40]. As other research has shown, food and nutrition security is the methodological and theoretical approach to food and nutrition policies and programs in Colombia since 2000 [11, 33]. Nonetheless, such an approach is reductionist as it is not supported by a research agenda that examines the legal, epistemological, and methodological limitations of using food security as the axis of food and nutrition programs and the discrepancies between food security-based approaches and the law framework on the RtAF [33]. Moreover, since food and nutrition security focuses on individual food access, it is ambiguous, as it does not indicate how to guarantee food access without harming the livelihoods of citizens, communities, and ecosystems [11, 41]**.** Our findings show two other ways by which this approach is reductionist. First, food and nutrition security is not a legal concept with correlative obligations, so it can be interpreted and implemented at the discretion of the organizations in charge. Second, food and nutrition security tends to be interpreted as the distribution of handouts to vulnerable individuals [10], which does not solve the structural causes of hunger and nutrition inequalities.

A handout-based approach to promote food and nutrition security prevails in multilateral agencies and national institutions [10–13]. The implementation of handout and nutritional supplementation programs is usually outsourced to tertiary providers of goods and services [12, 42]. Previous literature showed that outsourcing the implementation of government childhood programs creates a communication gap between the responsible organization and recipient families [32]. Our findings show that this gap can also occur in programs of multilateral agencies which are implemented by third parties. The gap impedes program accountability as program recipients cannot report problems, propose solutions, or output information to measure the impact of interventions [13, 17]. Furthermore, this and similar studies show insufficiency and obsolescence in food and nutrition information systems in Colombia [33–35], making it difficult to understand whether existing policies and programs serve to ensure the RtAF in all national territories.

This study also found that outsourcing the implementation of programs raises the risk of corruption. Previous literature identified corruption as a cause of inefficiency of food and nutritional policies in Colombia [31]**.** Our study sheds light on frequent forms of corruption related to outsourcing program implementation. Moreover, suppliers contracted to distribute food aid often exclude remote territories or send them inadequate aid, which inadvertently triggers illicit economies that thrive in conditions of hunger and poverty. These findings mirror studies in British Colonial Asia [43] and Brazil [44], showing that loss of agricultural income, food shortages, and unequal distribution of resources in areas of weak state presence are drivers of poverty, while poverty triggers different forms of crime [45, 46]. The problems described above reveal important gaps in food-security-assistance-based approaches to hunger and malnutrition relief. These approaches have characterized food and nutrition policies and programs in Colombia and many low- and middle-income countries [10–12, 23]. Our research suggests that formulating more effective food and nutrition programs requires a paradigm shift from seeing recipients as beneficiaries to understanding them as human rights holders and policy allies. This understanding implies generating participation mechanisms that integrate their voices in policy and program formulation, implementation, monitoring, and evaluation, including the design of indicators to longitudinally assess their impact and mobilization of resources to upscale community solutions to hunger and malnutrition.

No specific models were found for Colombia to prevent outsourcing policy and program implementation from hindering civic participation and to mobilize resources towards community solutions. However, a 2022 study systematically reviewed community-based participatory interventions to improve food and nutrition, finding that community-based participatory interventions have been applied in several low- and are middle-income countries and associated with improved nutritional, health, and food security outcomes. Furthermore, agricultural practices were improved, mainly after participatory models based on agroecology. Nonetheless, the review found insufficient evidence to support a specific type of participatory model to improve food and nutrition [47]. In addition, a recent study in Peru showed a high relevance of citizen participation in planning public strategies to reduce undernutrition [48], and in the United Kingdom, a model was developed to integrate children’s voices in research to design national food policies [49].

Peru and UK models are human rights based. Although a human rights-based policy approach has several advantages [50–52], there are potential limitations of this approach to address child hunger and malnutrition, including the potential for governments to claim economic and infrastructure difficulties to advancing economic development while simultaneously complying with their human rights obligations [50, 53]. Moreover, decision makers and influential actors who benefit directly or indirectly from current food-security-orientated measures and/or related outsourcing may not want to support or may even oppose a change of approaches, including initiatives to prevent corruption through citizen oversight. Recognizing this, the exploration of participatory models that adapt documented international best practices to the country context, identifying and addressing the potential limitations of human rights-based participatory approaches could be an opportunity for future research.

### Conclusion

This study canvassed the issue of policy and program adequacy to achieve the RtAF through a case study in Colombia, a review of the legal framework on the RtAF and a thematic analysis of 17 semi-structured interviews with experts. Findings from this research show that Colombia’s food-security-assistance-based policies and programs are failing to provide the RtAF for children. Given the importance of food for the attainment of all human rights, the need for an integrative approach to ensure children’s RtAF is urgent. This requires mechanisms to guarantee full citizen participation and accountability in policies and programs of government and international agencies. Like Colombia, many countries have food-security-assistance-based policies. Therefore, models to integrate citizen voices in food and nutrition decision making are an opportunity for future application and research insights from this study can serve as a baseline.

## Supplementary Material

Supplementary File S1 Guide for the semi-structured interviews (Colombia, 2022).
