# Supplementary material for: Barriers to Overcoming Child Hunger and Malnutrition: Applying a Human Rights Approach to Improve Policy and Action
Source: Int J Public Health. 2023 Aug 30;68:1605969. doi: 10.3389/ijph.2023.1605969 (PMC10498992; doi:10.3389/ijph.2023.1605969)
Supplement: Supplementary file 1 [file DataSheet1.PDF]

Interview Guide

The purpose of this interview is to compile information that may contribute to: (i) identifying the characteristics that efficient policies and programs should have to best prevent child hunger and malnutrition and realize children's right to food in Colombia; (ii) understanding the mechanisms that could explain the effectiveness of such policies and programs; (iii) identifying the main challenges to eradicate child hunger and malnutrition from a human rights perspective.

Questionnaire

Profile of the expert: ____

1. Do you consider that the high prevalence of child malnutrition in Colombia is associated to difficulties in the enforceability of the right to food?

2. Do you believe that food and childhood nutrition policies in Colombia are built through an approach that includes all stakeholders and civil society in the development of knowledge and solutions?

3. What are the current challenges to overcome child hunger and malnutrition in vulnerable in Colombia?

6. How should policies and programs be to efficiently tackle early malnutrition and progressively attain full observance of the right to adequate food of Colombian children?

7. Do you consider that there are specific vulnerabilities for certain groups of children? For example, differences between boys and girls, differences in family income, migrants, ethnicity.

8. How can international organizations better support civil society and local institutions to eradicate child hunger and malnutrition, protect and fulfill children's right to adequate food?
